# Supplementary material for: The structural and functional characterization of human RecQ4 reveals insights into its helicase mechanism
Source: Nat Commun. 2017 Jun 27;8:15907. doi: 10.1038/ncomms15907 (PMC5490261; doi:10.1038/ncomms15907)
Supplement: Supplementary Information [file ncomms15907-s1.pdf]

Type of file: PDF
Size of file: 0 KB
Title of file for HTML: Supplementary Information
Description: Supplementary figures and supplementary tables.

Type of file: PDF
Size of file: 0 KB
Title of file for HTML: Peer review file
Description:

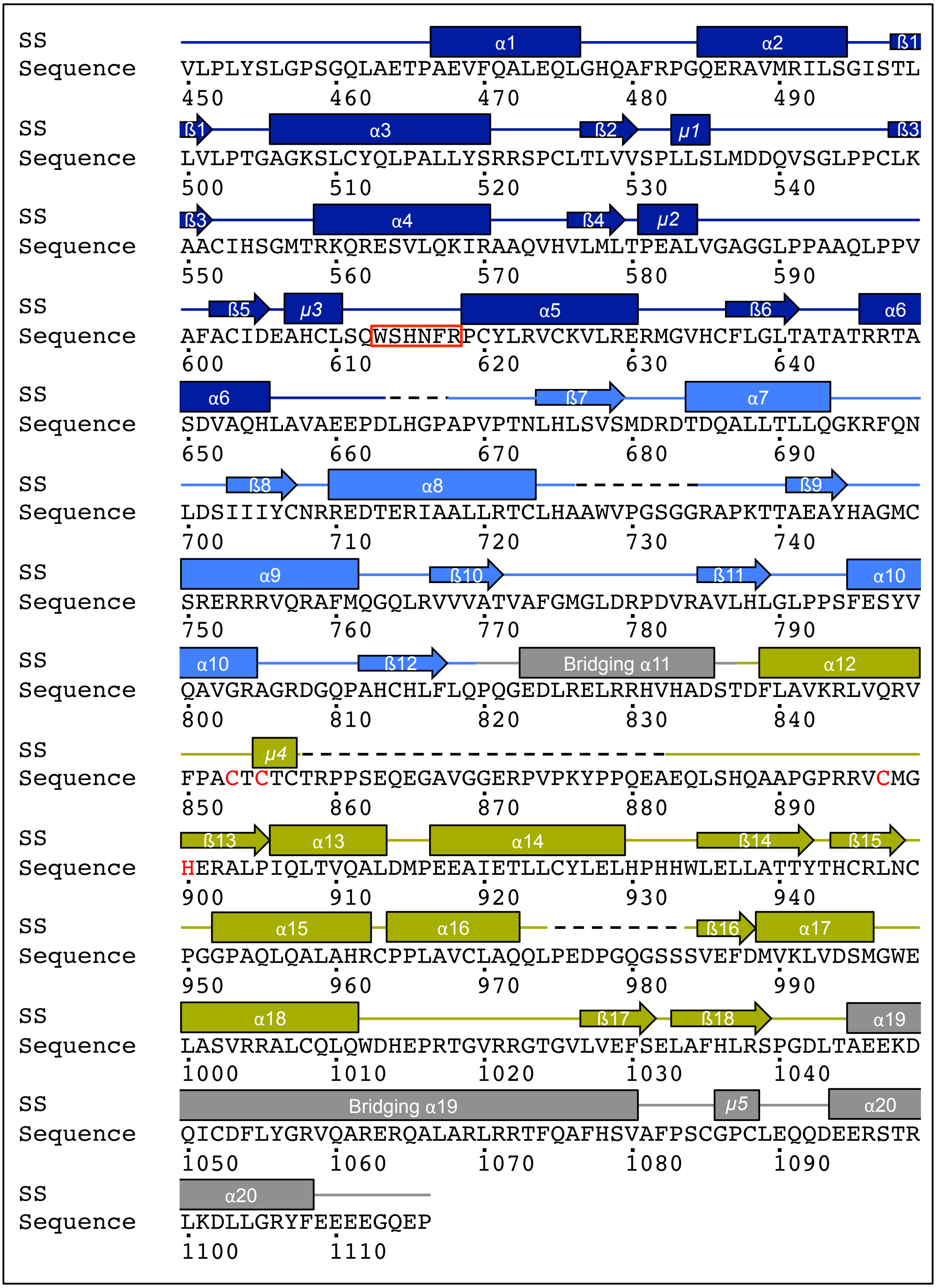

Supplementary Figure 1:
Annotation of secondary structure elements

Annotation of secondary structure elements within the RecQ4$^{427\text{-}1116}$ structure based on the DSSP algorithm. Domain color code as in Figure 1. Briefly: dark blue – HD1, light blue – HD2, olive – R4ZBD, gray – bridging helices. Helices are depicted as boxes. β-strands are represented by arrows. 3-10-helices are indicated by the symbol μ in italic. The ARL-sequence is highlighted by a red box and the Zn^{2+}-coordinating residues are depicted in red. Regions which are not resolved in the structure are illustrated by dashed lines.

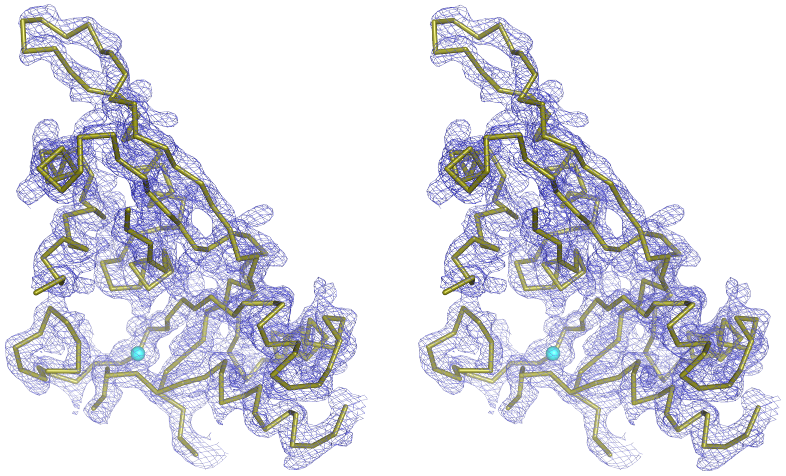

Supplementary Figure 2:
Stereo-view of the R4ZBD

Stereo view of the the RecQ4-zinc-binding domain (R4ZBD) depicted in ribbon representation (Cα trace). The zinc ion is shown as a cyan sphere. The $2F_o$-F_c omit electron density map is shown at 1σ (blue mesh).

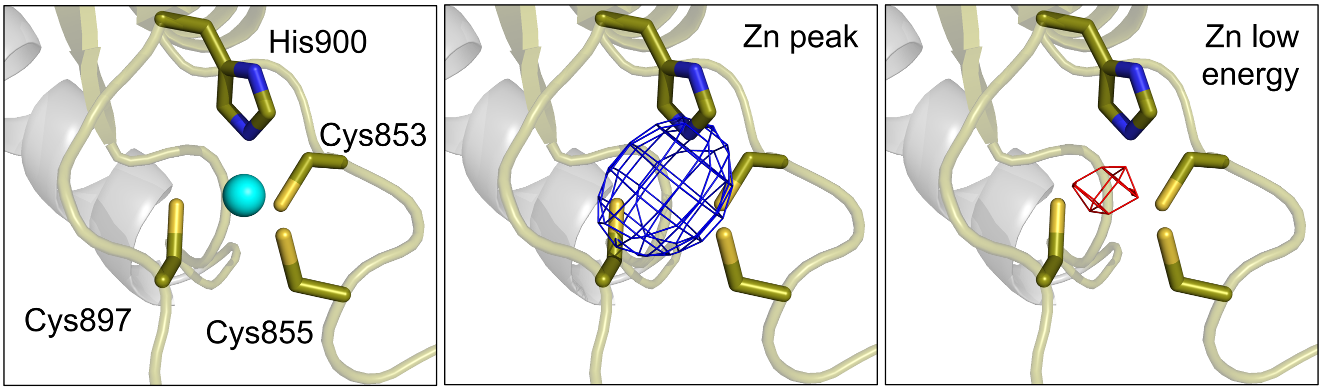

Supplementary Figure 3:
Confirmation of the bound Zn^{2+} ion by anomalous difference maps

Position of the Zn^{2+} ion within the R4ZBD. Zn^{2+}-coordinating residues are illustrated and depicted in stick representation. Left: Zn^{2+} ion in sphere representation. Middle: Anomalous electron density (blue mesh) at the Zn-peak-energy (1.2823 Å). Right: Anomalous electron density (red mesh) at low-energy (1.2831 Å). Both maps are shown at 10 σ. While there is a strong anomalous signal at the Zn-peak energy, the signal is strongly reduced for the energy below the absorption peak, confirming the identity of a Zn^{2+} ion.

No.	PDB / chain	Z-value	RMSD	align	% id.	Protein class*
1	2fmy-A	6.7	2.8 Å	71	15	DNA binding protein
2	3h0d-A	6.6	2.6 Å	67	4	Transcription / DNA
3	1o57-A	6.3	2.5 Å	67	9	DNA binding protein
4	3iwz-D	6.3	2.8 Å	65	14	Transcription
5	4r6i-A	6.1	2.9 Å	79	11	Transcription
6	2oz6-A	6.1	2.2 Å	62	13	DNA binding protein
7	1ylf-C	5.9	2.8 Å	79	9	Transcription regul.
8	5e44-A	5.6	3.1 Å	70	14	Transcription
9	2xhk-B	5.6	2.7 Å	67	15	Transcription
10	2z9o-A	5.6	3.2 Å	72	6	Replication / DNA

*according to the PDB classification

Supplementary Table 1:
DALI results for the entire R4ZBD

Top 10 structural homologs of the DALI PDB search for the entire R4ZBD (aa 863-1060). Matches were obtained from the PDB90 database.

No.	PDB / chain	Z-value	RMSD	align	% id.	Protein class*
1	5d6e-A	4.3	3.0 Å	59	19	Hydrolase
2	3ife-A	3.8	3.2 Å	61	8	Hydrolase
3	3h0d-A	3.8	2.5 Å	49	10	Transcription / DNA
4	4ney-B	3.8	3.6 Å	59	8	*De novo protein*
5	3rir-A	3.7	2.4 Å	47	9	Ligase
6	2mzj-A	3.7	3.1 Å	61	10	RNA Binding Protein
7	1u8s-B	3.7	2.9 Å	54	6	Transcription
8	3mgj-A	3.6	3.4 Å	55	4	*Unknown function*
9	3mah-A	3.5	2.4 Å	49	8	Transferase
10	2nzc-D	3.5	2.8 Å	52	13	*Unknown function*

*according to the PDB classification

Supplementary Table 2:
DALI results for the top half R4ZBD

The top 10 structural homologs of the DALI PDB search for the top half R4ZBD (aa 942-1032). Matches were obtained from the PDB90 database.

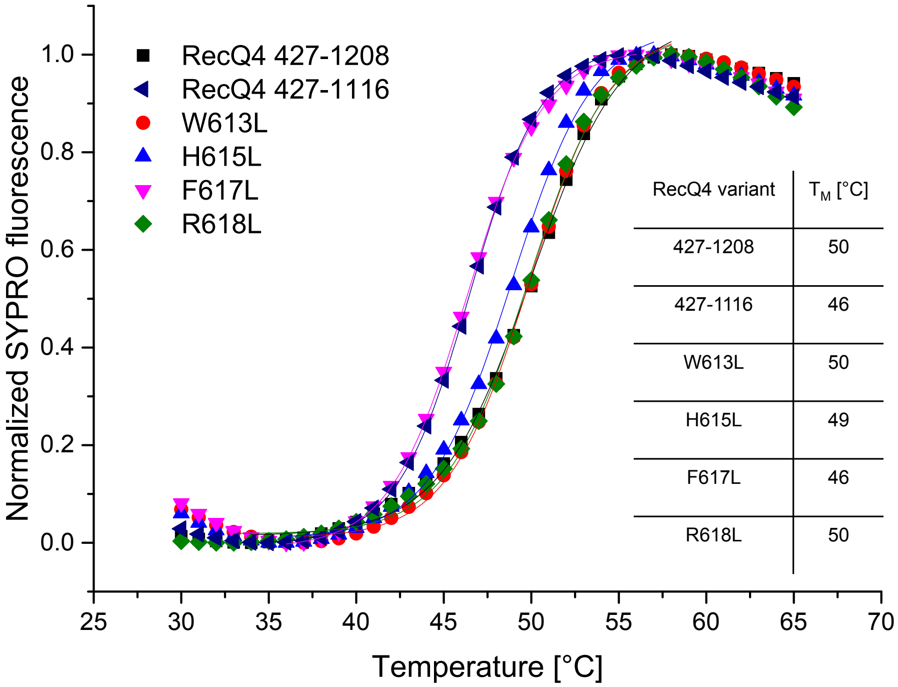

RecQ4 variant	T_M [°C]
427-1208	50
427-1116	46
W613L	50
H615L	49
F617L	46
R618L	50

Supplementary Figure 4:
Thermal stability analysis (Thermofluor assay) of the RecQ4 ARL variants

Melting curves for all tested RecQ4 ARL variants display the gradual unfolding with increasing temperature. The melting temperature at a 50% unfolded state is represented by the T_M value. The melting curves indicate that all RecQ4 variants are properly folded as all variants display regular denaturing curves. The ARL variant F617L and $RecQ4^{427-1116}$ are less stable compared to other RecQ4 variants, indicated by their decreased T_M value.

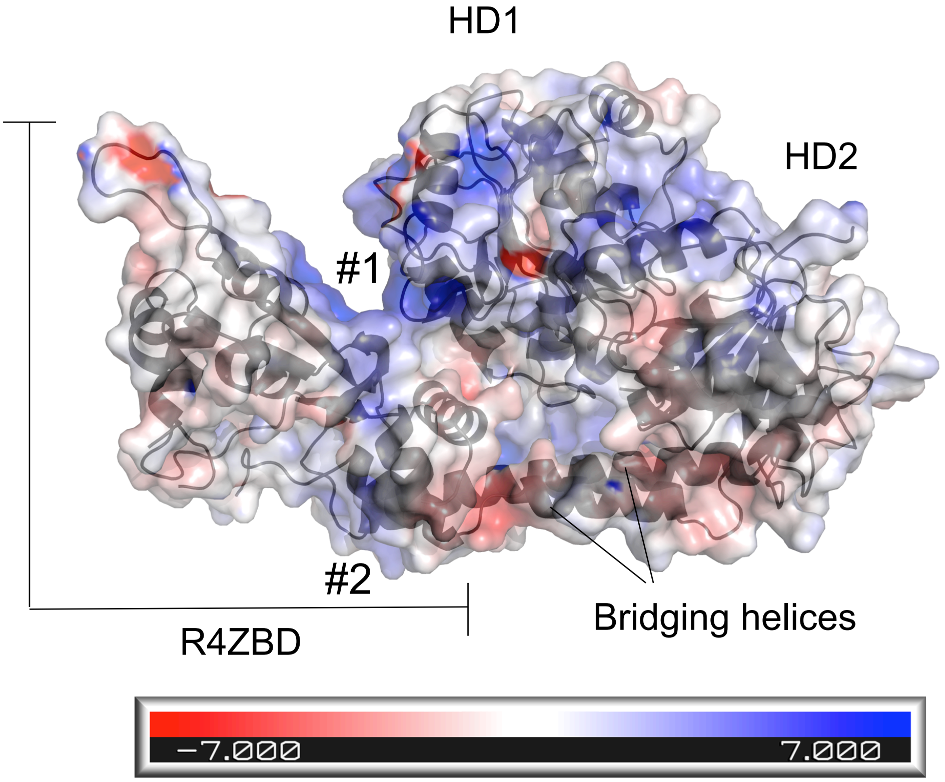

Supplementary Figure 5:
Electrostatic surface potential map of the RecQ4$^{427\text{-}1116}$ model

The electrostatic surface potential was calculated using the APBS plugin in pymol. The orientation of the model is similar to the orientation in Figure 1b (left). Electronegative areas are shown in red, while the blue color illustrates an electropositive surface potential. The RecQ4$^{427\text{-}1116}$ model features a large electropositive surface area directly between the R4ZBD and the HD1 (indicated as #1). A second electropositive patch is located at the bottom of the lower R4ZBD half (depicted as #2), which features the standard three-stranded WH-like DNA binding motif as shown in Figure 2c (left).

Constructs	Forward Primer (5’-3’)	Reverse Primer (5’-3’)
Vector linearization	GGATCCGAATTCGAGCTC	GGGCCCCTGGAAC
427-1208	TCTGTTCCAGGGGCCCATGGCTGTTGGGCCTGAG	GCTCGAATTCGGATCCTCAGCGGGCCACC
427-1116	TCTGTTCCAGGGGCCCATGGCTGTTGGGCCTGAG	GCTCGAATTCGGATCCTCACGGCTCCTGCCCTTC
427-1090	CGGGCCCTGCCTGGAGTGAGGATCCGAATTCGAGCTCCGTCGACAAGC	CTCCAGGCAGGGCCCGCAG
del944-1032	ACCACCTATACCGGAGGTGAGCTGGCCTTCCACCTTCGCA	AGGCCAGCTCACCTCCGGTATAGGTGGTCGCCAGCAGCTC
K508A	GGTGCCGGCGCGTCCCTGTGCTACCAG	CGGATGTCCACGGCCGCGCAGGGACAC
D605A	GCTTTTGCCTGCATTGCTGAGGCCCACTGCCTC	AGGCAGTGGGCCTCAGCAATGCAGGCAAAAGC
R894A/R895A	CAAGCAGCCCCAGGACCCGCGGCGGTCTGCATGGGCCATGAG	TCATGGCCCATGCAGACCGCCGCGGGTCCTGGGGCTGCTTGG
K1048A	CCGCTGAGGAGGCGGACCAGATATG	ATATCTGGTCCGCCTCCTCAGCGGT
R844A	TGGCTGTGAAGGCGCTGGTACAGCG	GCTGTACCAGCGCCTTCACAGCCAG
R848A*	GTGAAGGCGCTGGTACAGGCGGTGTTCCCAGCCTGC	GCAGGCTGGGAACACCGCCTGTACCAGCGCCTTCAC
K843A**	CGGACTTCCTGGCTGTGGCGGCGCTGGTACAGG	CCTGTACCAGCGCCGCCACAGCCAGGAAGTCCG
R844E***	CTGGCTGTGGCGGAGCTGGTACAGGCGG	CCGCCTGTACCAGCTCCGCCACAGCCAG
W613L	CTGCCTCTCCCAGCTGTCCCACAACTTC	GAAGTTGTGGGACAGCTGGGAGAGGCAG
H615L	CCCAGTGGTCCCTGAACTTCCGGCCC	GGGCCGGAAGTTCAGGGACCACTGGG
F617L	GTCCCACAACTTACGGCCCTGCTAC	GTAGCAGGGCCGTAAGTTGTGGGAC
R618L	CCCACAACTTCCTGCCCTGCTACCTG	CAGGTAGCAGGGCAGGAAGTTGTGGG

* introduced in the R844A template

** introduced in the R843A/R848A template

*** introduced in the K843A/R844A/R848A template

Supplementary Table 3:
Primer sequences used for cloning and mutagenesis of the RecQ4 variants of this study
